# Accuracy of Leg Length and Offset Restoration in Femoral Pinless Navigation Compared to Navigation Using a Fixed Pin during Total Hip Arthroplasty

**DOI:** 10.1155/2018/1639840

**Published:** 2018-06-25

**Authors:** Markus Weber, Max Thieme, Moritz Kaiser, Florian Völlner, Michael Worlicek, Benjamin Craiovan, Joachim Grifka, Tobias Renkawitz

**Affiliations:** ^1^Department of Orthopedic Surgery, Regensburg University Medical Center, Kaiser-Karl V.-Allee 3, 93077 Bad Abbach, Germany; ^2^Department of Trauma Surgery, Regensburg University, Medical Center, Franz-Josef-Strauß-Allee 11, 93053 Regensburg, Germany

## Abstract

Equalization of biomechanical differences is a major goal in total hip arthroplasty (THA). In the current study we compared the accuracy of restoring leg length and offset using imageless navigation with an osseous fixed pin to a femoral pinless device in 97 minimally invasive THAs through an anterolateral approach in the lateral decubitus position. Leg length and offset differences were evaluated on magnification-corrected radiographs by a blinded observer. A postoperative mean difference of -0.9 mm (95% CI -2.8 mm to 1.1 mm,* p* = 0.38) between pinless navigation and navigation with a fixed pin was observed for leg length and that of -2.4 mm (95% CI -3.9 mm to -0.9 mm,* p* = 0.002) was observed for offset, respectively. The number of patients with a residual difference below 5 mm after THA was higher if using a fixed pin than in pinless navigation for both leg length (98.2%, 54/55 to 50.0%, 21/42,* p* < 0.001) and offset (100.0%, 55/55 to 71.4%, 30/42,* p* < 0.001). Imageless navigation is a feasible method in intraoperative control of leg length and offset in minimally invasive THA. The use of pins fixed to the bone has a higher precision than pinless devices. This trial is registered with DRKS00000739.

## 1. Introduction

Accuracy in restoration of biomechanics such as leg length and offset is crucial in total hip arthroplasty (THA) [1]. Both leg length and offset discrepancy after THA are a major source of patient dissatisfaction and finally litigation [2]. Failure in biomechanical restoration is associated with gait asymmetry and lower back pain [3, 4]. Even small differences in leg length and offset above 5 mm lead to alterations in gait kinematics as measured by gait analysis [5]. Leg length discrepancies after THA have been reported to correlate with abnormal force transmission, aseptic loosening, and early revision surgery [1, 6]. Likewise, a weak restoration of hip offset is related to hip instability, impingement, and polyethylene wear [7–9].

In clinical practice the orthopaedic surgeon usually aims to improve accuracy in biomechanical restoration by preoperative planning and intraoperative use of mechanical devices, alignment guides, pins, rulers, or fluoroscopy [10, 11]. However, these measurements are susceptible to error due to potential changes in leg position between measurements. Small changes in abduction/adduction of 5 mm lead to an error of 8 mm in leg length estimation [12]. Over the recent two decades the technical progress has opened novel possibilities by the development of computer assisted navigation. Imageless navigation systems without the need for preoperative or intraoperative image acquisition and exposure to radiation have been reported to increase the accuracy of positioning the acetabular component [13]. This technique also harbours the possibility of controlling leg length and offset changes during THA [14]. To reduce the risk of pin infection and fractures due to the bony fixation of reference markers [15], novel pinless navigation devices have been developed for achieving appropriate LL and OS values [16]. However, direct translatory and rotational variations between the pinless array and the femoral bone up to 8 mm in translation and 9° in rotation are associated with the risk of potential error during measurements [17]. Although the clinical accuracy of fixed-pin [14] and pinless [18] computer assisted navigation has been investigated independently in previous studies, no study has directly compared the two different techniques with each other.

In the current study we aimed to investigate the (1) accuracy, (2) precision, and (3) number of outliers above 5 mm in restoration of leg length and offset between fixed-pin and pinless navigated minimally invasive THA.

## 2. Patients and Methods

In the course of two prospective clinical trials (DRKS00000739) patients underwent navigated THA at our University Medical Center. After authorization by the Institutional Ethical Board (Nos. 10-121-0263, 10-101-0121) written informed consent was obtained. This study is a secondary analysis of the data collected in the two original trials. The first study evaluated intraoperative leg length and offset changes in relation to 3D-CT using a pinless reference marker [18]. The pinless marker had been previously validated in an in vitro experiment using human specimens [19]. This method ensures that the leg is placed in the same orientation relative to the pelvis before and after reconstruction and eliminates the need to calculate the center of the hip or to establish a femoral coordinate system. In contrast, the second study compared the accuracy in leg length and offset restoration between intraoperative fluoroscopy and navigated THA with a reference marker fixed to the bone [14]. The study designs, patient recruitment, and inclusion/exclusion criteria are detailed in Renkawitz et al. [18] and Weber et al. [14]. In the current study we now performed a direct comparison between the pinless and fixed-pin navigation technique using these two cohorts in terms of the accurate restoration of leg length and offset in minimally invasive THA.

**Figure 1:**
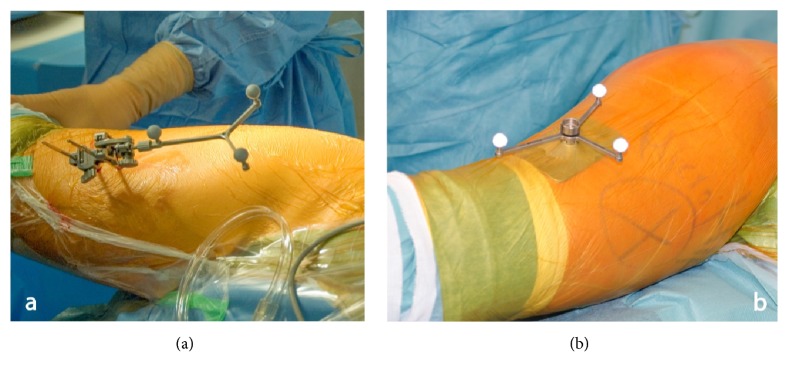
Comparison between the standard fixation of the femoral marker using a fixed pin (a) and the pinless device secured with incision foil (b).

**Figure 2:**
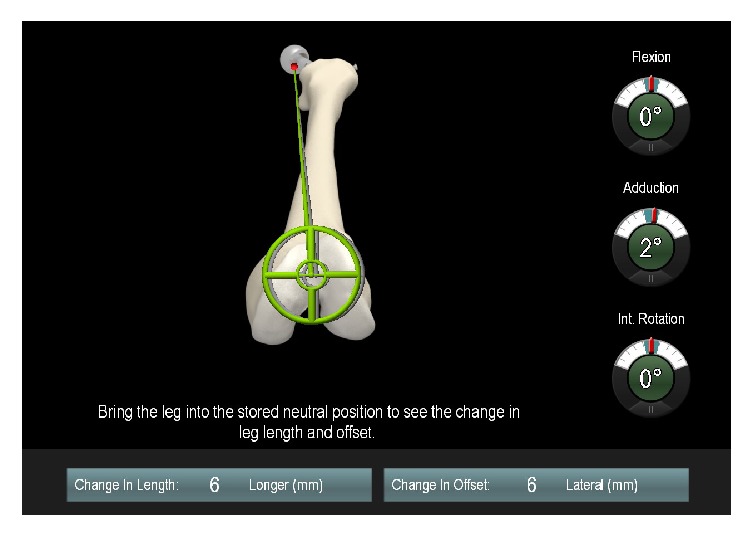
Intraoperative measurement of leg length and offset change in a neutral leg position using imageless navigation as displayed on the screen.

Prior to surgery, restoration of leg length and offset was templated on digital AP radiographs of the pelvis with the help of digital planning software (MediCAD, Hectec, Germany) for all patients. The radiographic magnification was corrected using a scaling object of known diameter. With the unaffected contralateral side serving as a reference, leg length and offset differences were corrected. All operations were performed in the lateral decubitus position through a minimally invasive anterolateral approach to the hip [20]. Press-fit components (Pinnacle, DePuy, Warsaw, IN, USA) and cement-free hydroxyapatite-coated stems (Corail, DePuy) were used. A brief description of the navigated pinless and fixed-pin surgical procedure is presented below. For both techniques as part of the navigation data entry, two connected K-wires (3.2 mm in diameter) were inserted in the ipsilateral iliac wing first. A dynamic reference array then was connected to these wires. On the femoral side the fixation differed between the pinless and fixed-pin technique. Whereas two K-wires were inserted in the ventrolateral one-third of the distal femur for bony fixation of the dynamic reference array in the fixed-pin group, the pinless referencing device (pinless array, Brainlab, Feldkirchen, Germany) was positioned laterally on the distal third and secured with an incision foil (Opsite, Smith and Nephew, Marl, Germany). The pinless array consists of a plate, which mimics the anatomic shape of the soft tissue; the dynamic reference array itself is rigidly attached to this plate by a screw (Figure 1). In both groups a preoperative neutral reference position of the leg was defined by holding it in approximately zero degree of flexion, abduction, and rotation. The navigation system stored the relative orientation (transformation) between the femur and pelvis dynamic reference array according to this position. After inserting the trial and final implants and hip reduction, the initial neutral reference position was reproduced. The navigation system guided the surgeon by showing the deviation between the current and the initial neutral reference alignment. In the pinless cohort a small reference screw was fixed into the greater trochanter as an additional bony reference landmark. After inserting the implants, the initial neutral reference position was reproduced and the trochanteric reference screw was reregistered. Leg length and offset change as presented on the screen were stored three times for reproducibility and the mean of these measurements was considered the true leg length and offset change (Figure 2). All surgeons aimed to restore leg length and offset according to the preoperative plan.

**Figure 3:**
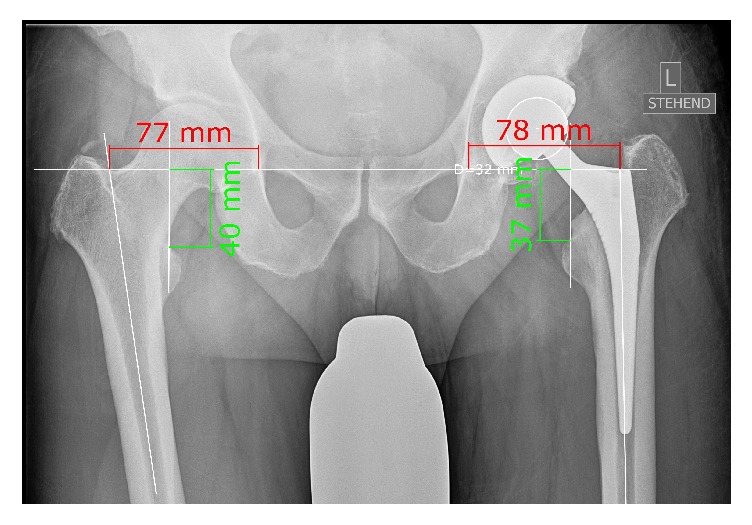
Postoperative assessment of residual leg length and offset differences in relation to the contralateral side on postoperative radiographs.

Postoperatively leg length and offset differences were evaluated on standardized digital AP radiographs of the pelvis using the same digital planning software as for templating. Magnification was normalized using the known size of the prosthesis head. Measurements were performed as previously described [21]. In brief, leg length was obtained by drawing a line through the inferior aspects of the teardrops (interteardrop line or Koehler line) and measuring the distance to the superior margin of the lower trochanter [10, 11]. Global offset was defined as the distance from the center of rotation of the femoral head to the teardrop along the transteardrop line touching the inferior margins of the teardrop [22]. To maximize accuracy, the distances between the long axis and the outer contours of the femur were checked carefully on the radiographs. The axes were placed in a way that the distances between preoperative and postoperative radiographs matched in the proximal and the more distal parts of the femoral canal. All postoperative radiographic measurements were performed by a blinded observer (MW) independently of the surgical team (Figure 3).

In total, records of 97 patients (55 fixed-pin and 42 pinless navigated THAs) were included for final analysis. Anthropometric characteristics of the two groups are shown in Table 1. The relative accuracy was defined on radiographs as the relative postoperative difference between the operated and the unaffected contralateral side for leg length and offset, respectively. Precision was defined as the absolute postoperative deviation of leg length and global offset regardless of lengthening or shortening of leg length and offset throughout the THA. Since biomechanical discrepancies above five millimetre are associated with altered gait kinematics, a postoperative leg length or offset inequality greater than 5 mm was regarded as outlier [5]. For statistical analysis, continuous data for navigation are presented as mean ± standard deviation. Differences between the fixed-pin and pinless navigation are presented as mean and 95% confidence interval of the difference (95% CI). Group comparisons were performed using two-sided* t*-tests for the normally distributed variables leg length and offset differences and Mann–Whitney* U* tests for the variables absolute leg length and absolute offset differences (precision) due to nonnormal distribution, respectively. Absolute and relative frequencies were given for categorical data and compared between study groups using Fisher's exact tests. Due to the analysis of two variables (leg length and offset) all hypotheses were tested on a Bonferroni adjusted, two-sided 5%/2 = 2.5% significance level. IBM SPSS Statistics 22 (SPSS Inc, Chicago, IL, USA) was used for analysis.

## 3. Results

**Figure 4:**
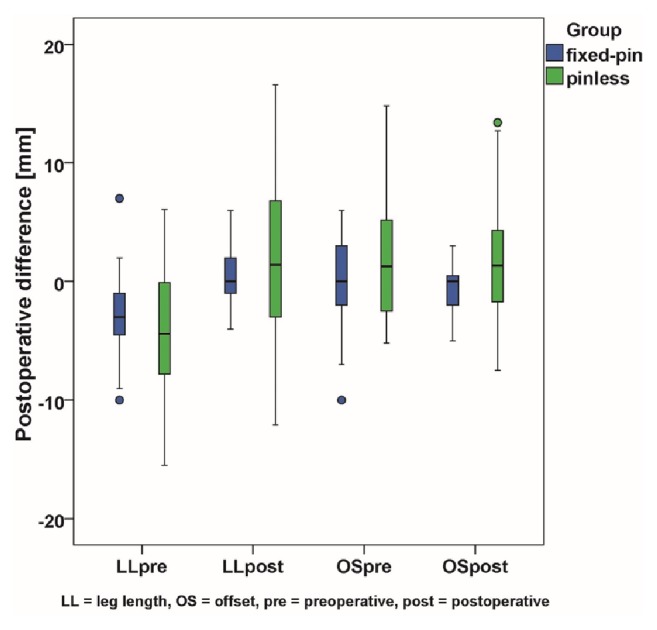
Pre- and postoperative leg length and offset differences between imageless navigation using a fixed reference marker and the pinless device.

Analysing the accuracy in restoration of leg length between navigation using a fixed pin and the pinless technique we observed a postoperative mean difference of -0.9 mm (95% CI -2.8 mm to 1.1 mm,* p* = 0.38) between the two navigation methods in minimally invasive THA. Correspondingly, for offset restoration we found a postoperative mean difference between fixed-pin and pinless navigation of -2.4 mm (95% CI -3.9 mm to -0.9 mm,* p* = 0.002). In the preoperative situation leg length and offset differences were comparable between the two groups with a mean difference of 1.6 mm (95% CI -0.2 mm to 3.5 mm,* p* = 0.09) for leg length and of -1.7 mm (95% CI -3.5 mm to 0.5 mm,* p* = 0.06) for offset, respectively. Pre- and postoperative leg length and offset discrepancies in the group with fixed pins and the pinless group are shown in Figure 4.

Measuring precision by absolute deviations in biomechanical restoration we found a mean absolute leg length difference of 1.7 mm ± 1.3 mm for navigation using fixed pins compared to 5.5 mm ± 3.9 mm (*p* < 0.001) for the pinless device. Similarly, global offset was restored with an absolute mean of 1.4 mm ± 1.3 mm for the fixed-pin technique and 4.2 mm ± 3.5 mm (*p* < 0.001) for pinless navigation.

**Figure 5:**
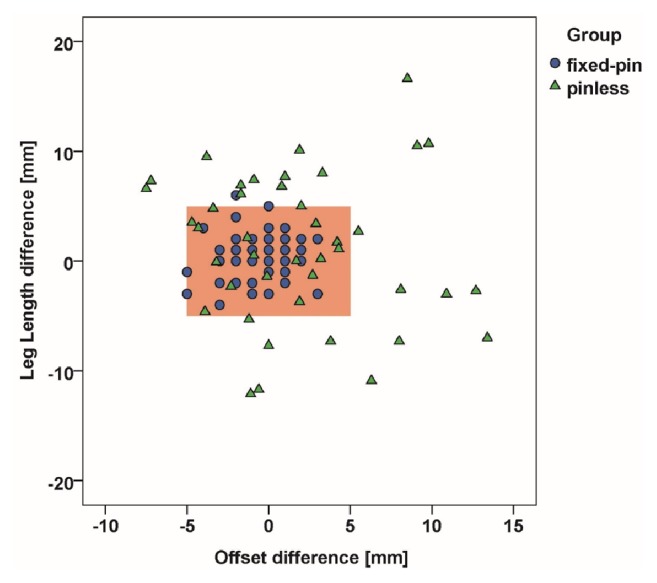
Distribution of residual postoperative leg length and offset differences of navigation using a fixed pin in relation to femoral pinless navigation.

Researching into outliers above 5 mm, 98.2% (54/55) of patients with fixed pins and 50.0% (21/42) of pinless patients were inside the tolerance limit of 5 mm in terms of successful leg length restoration (*p* < 0.001). Global offset of 100.0% (55/55) of fixed-pin navigated patients and that of 71.4% (30/42) of pinless navigated patients did not exceed the 5 mm benchmark (*p* < 0.001, Figure 5).

## 4. Discussion

Failure in restoration of leg length and offset is a frequent problem of functional impairment and patient dissatisfaction after THA [6]. Therefore, methods to intraoperatively control leg length and offset changes during THA are of high interest to the orthopaedic community. Computer assisted navigation is one option to assess biomechanical restoration during surgery independently of leg position [13]. In the present study we aimed to compare the accuracy of restoring leg length and offset during navigation-guided THA between a technique using a bony fixed reference marker and a pinless device without the need of femoral intraosseous pins. We found a good accuracy with mean differences of 1 mm between the two fixation methods. However, the absolute precision was higher in the group using a fixed pin than in the pinless group for both leg length (1.7 mm to 5.5 mm) and offset (1.4 mm to 4.2 mm). Similarly, the number of outliers with postoperative differences above 5 mm was lower in the fixed-pin group with lower than 5 % compared to the pinless group with up to 50 %.

There are several limitations of this study. In this study two different cohorts from two prospective trials were compared with each other. Therefore, there was no possible randomization for pinless navigation versus navigation using a fixed pin. Due to the lack of randomization potential bias cannot be ruled out. However, patients' characteristics were comparable between the two groups. Similarly, there were no relevant differences in preoperative leg length and offset discrepancies between the pinless and fixed-pin group. In terms of evaluating leg length and offset radiographic measurements on anteroposterior radiographs of the pelvis and femur they are susceptible to error since horizontal dimensional parameters are influenced by variations in positioning of the pelvis relative to the plane of the film and the divergence of the X-ray beams [23]. The reliability of these measurements is further reduced by the influence of pelvic tilt and rotation [24]. To improve accuracy, patients were placed in a standardized position and we used a magnification marker and digital planning software for our radiographic analysis. As recommended in literature [25] the interteardrop line was favoured over the biischial line for measurements because of its diminished susceptibility to pelvic rotation. The radiographic measurement workflow applied in this study had been evaluated in a recent study. Radiographic measurements on anteroposterior pelvic radiographs showed a high accuracy of 1.0 mm ± 2.0 mm compared to 3D-CT for leg length and of 0.6 mm ± 3.6 mm for global offset, respectively. In contrast, measurements of femoral offset on plain radiographs showed a higher deviation compared to 3D-CT [21]. This is the reason why we concentrated on leg length and global offset evaluation in this study and omitted femoral offset. The use of navigation devices has three general limitations. First, navigation computers are susceptible to crashing, which happened once during our study. Therefore, surgeons using navigation always need to be aware of potential malfunction of the system and should be able to continue surgery without computer assistance at any time. Second, the use of navigation generally increases operation time of about 15 minutes due to registration and intraoperative measurements [26]. Third, purchase and service of navigation systems are of significant financial expense.

The use of femoral pinless navigation has four general limitations: First, the workflow does not offer a navigated preparation of the femoral medullary canal and/or an intraoperative control of hip kinematics or impingement. Second, an additional point in the proximal part of the femur has to be referenced before and after component placement by inserting a small screw in the trochanteric region. Third, it remains still necessary to insert pins into the iliac crest to attach a pelvic dynamic reference array. This harbours the risk of injury, infection, or even fracture [15]. Last, shifting of the pinless array may result in measurement inaccuracies. A previous study has reported movements of the pinless array during hip movement up to 8 mm in translation and 9° in rotation [17]. Although measurement algorithms in which the calculation is based on a defined measurement position and a specific realignment of the leg are able to reduce this inaccuracy, slight movements of the pinless reference array might be one reason for the higher precision of leg length and offset measurement if using a marker fixed to the femoral bone.

In answer to the first question of this study we found a mean inaccuracy below 2 mm for both the pinless navigation and navigation with a fixed pin in restoring leg length and offset. This is in line with literature reporting a mean difference below 1 mm for leg length restoration and below 2 mm for offset equalization in navigated THA [22]. Differences below 2 mm seem barely measurable. In this context imageless navigation regardless of the type of marker fixation seems in general a reliable tool in restoring biomechanics during THA. However, we observed a lower precision for pinless navigation in the current study. Whereas the absolute postoperative mean differences were still below 2 mm in the navigation group using a bony fixed reference marker the absolute deviation from the intraoperative aim to equalize leg length and offset was about 5 mm in the pinless group. In order to assess the clinical relevance of this higher precision for navigation using a fixed femoral reference array we researched into the number outliers. The definition of outlier in terms of leg length and offset differences is controversial in literature. The maximum tolerable difference after THA varies between 10 mm and 5 mm for leg length [4, 10, 27] and offset [7, 22]. Based on gait analysis which observed alterations in gait kinematics for leg length and offset differences above 5 mm [5], we defined a successful restoration zone for leg length and offset differences below 5 mm after THA. Using this strict benchmark, we observed a higher number of outliers in the pinless group compared to navigation using a fixed marker. This indicates clinically relevant differences between the two fixation methods in navigated minimally invasive THA. This might be due to translatory and rotational variations between the pinless array and the femoral bone which have been reported up to 8 mm in translation and 9° in rotation [17]. In contrast to a previous in vitro study showing a high reliability for leg length and offset measurement using a pinless femoral reference array with differences below 1 mm compared to 3D-CT [16], our study was not able to fully support these results in a clinical setting.

## 5. Conclusions

In conclusion imageless navigation in minimally THA represents a feasible assistance in controlling leg length and offset intraoperatively. Exact preoperative planning and accurate assessment of leg length and offset differences prior to THA are crucial for successful biomechanical restoration. For intraoperative assistance navigation systems with a pin fixed to the bone show a higher precision than a femoral pinless device.

## Figures and Tables

**Table 1:** Anthropometric characteristics of the study group.

	Fixed Pin	Pinless
Probands (number)	55	42
Age (years)	62.4 ± 7.6	65.8 ± 6.1
Sex (men/women)	27/28	19/23
BMI (kg/m2)	27.7 ± 4.1	26.7 ± 4.2
Treatment side (right/left)	27/28	21/21
Kellgren-Lawrence score	8 (6-10)	9 (5-10)
Operative time (min)	77 (51–126)	75 (50-116)

## Data Availability

The data used to support the findings of this study are available from the corresponding author upon request.

## References

[1] Konyves A., Bannister G. C. (2005). The importance of leg length discrepancy after total hip arthroplasty. *The Journal of Bone & Joint Surgery (British Volume)*.

[2] Hofmann A. A., Skrzynski M. C. (2000). Leg-length inequality and nerve palsy in total hip arthroplasty: A lawyer awaits!. *Orthopedics*.

[3] Gurney B., Mermier C., Robergs R., Gibson A., Rivero D. (2001). Effects of limb-length discrepancy on gait economy and lower-extremity muscle activity in older adults. *The Journal of Bone & Joint Surgery*.

[4] Friberg O. (1983). Clinical symptoms and biomechanics of lumbar spine and hip joint in leg length inequality. *The Spine Journal*.

[5] Renkawitz T., Weber T., Dullien S. (2016). Leg length and offset differences above 5 mm after total hip arthroplasty are associated with altered gait kinematics. *Gait & Posture*.

[6] Röder C., Vogel R., Burri L., Dietrich D., Staub L. P. (2012). Total hip arthroplasty: Leg length inequality impairs functional outcomes and patient satisfaction. *BMC Musculoskeletal Disorders*.

[7] Little N. J., Busch C. A., Gallagher J. A., Rorabeck C. H., Bourne R. B. (2009). Acetabular polyethylene wear and acetabular inclination and femoral offset. *Clinical Orthopaedics and Related Research*.

[8] Spalding T. J. (1996). Effect of femoral offset on motion and abductor muscle strength after total hip arthroplasty.. *The Journal of Bone & Joint Surgery (British Volume)*.

[9] Sakalkale D. P., Sharkey P. F., Eng K., Hozack W. J., Rothman R. H. (2001). Effect of femoral component offset on polyethylene wear in total hip arthroplasty. *Clinical Orthopaedics and Related Research*.

[10] Ranawat C. S., Rao R. R., Rodriguez J. A., Bhende H. S. (2001). Correction of limb-length inequality during total hip arthroplasty. *The Journal of Arthroplasty*.

[11] Maloney W. J., Keeney J. A. (2004). Leg length discrepancy after total hip arthroplasty. *The Journal of Arthroplasty*.

[12] Sarin V. K., Pratt W. R., Bradley G. W. (2005). Accurate femur repositioning is critical during intraoperative total hip arthroplasty length and offset assessment. *The Journal of Arthroplasty*.

[13] Renkawitz T., Tingart M., Grifka J., Sendtner E., Kalteis T. (2009). Computer-assisted total hip arthroplasty: coding the next generation of navigation systems for orthopedic surgery. *Expert Review of Medical Devices*.

[14] Weber M., Woerner M., Springorum R. (2014). Fluoroscopy and imageless navigation enable an equivalent reconstruction of leg length and global and femoral offset in THA.. *Clinical Orthopaedics and Related Research*.

[15] Jung H., Jung Y., Song K., Lee J., Park S. (2007). Fractures Associated with Computer-Navigated Total Knee Arthroplasty. *The Journal of Bone and Joint Surgery-American Volume*.

[16] Renkawitz T., Schuster T., Grifka J., Kalteis T., Sendtner E. (2010). Leg length and offset measures with a pinless femoral reference array during THA. *Clinical Orthopaedics and Related Research*.

[17] Renkawitz T., Wegner M., Gneiting S. (2010). Experimental validation of a pinless femoral reference array for computer-assisted hip arthroplasty. *Journal of Orthopaedic Research*.

[18] Renkawitz T., Sendtner E., Schuster T., Weber M., Grifka J., Woerner M. (2014). Femoral pinless length and offset measurements during computer-assisted, minimally invasive total hip arthroplasty. *The Journal of Arthroplasty*.

[19] Renkawitz T., Gneiting S., Schaumburger J. (2010). In-vitro investigation of a noninvasive referencing technology for computer-assisted total hip arthroplasty. *Orthopedics*.

[20] Michel M. C., Witschger P. (2006). MicroHip: a minimally invasive procedure for total hip replacement surgery. A modified Smith-Peterson approach. *Interactive Surgery*.

[21] Weber M., Woerner M. L., Springorum H.-R., Hapfelmeier A., Grifka J., Renkawitz T. F. (2014). Plain Radiographs fail to reflect femoral offset in total Hip Arthroplasty. *The Journal of Arthroplasty*.

[22] Dastane M., Dorr L. D., Tarwala R., Wan Z. (2011). Hip offset in total hip arthroplasty: Quantitative measurement with navigation. *Clinical Orthopaedics and Related Research*.

[23] Varghese B., Muthukumar N., Balasubramaniam M., Scally A. (2011). Reliability of measurements with digital radiographs - A myth. *Acta Orthopædica Belgica*.

[24] Rubin P. J., Leyvraz P. F., Aubaniac J. M., Argenson J. N., Esteve P., De Roguin B. (1992). The morphology of the proximal femur: a three-dimensional radiographic analysis. *The Journal of Bone & Joint Surgery (British Volume)*.

[25] Meermans G., Malik A., Witt J., Haddad F. (2011). Preoperative radiographic assessment of limb-length discrepancy in total hip arthroplasty. *Clinical Orthopaedics and Related Research*.

[26] Renkawitz T., Wörner M., Sendtner E., Weber M., Lechler P., Grifka J. (2011). Principles and new concepts in computer-navigated total hip arthroplasty. *Der Orthopäde*.

[27] Woolson S. T., Hartford J. M., Sawyer A. (1999). Results of a method of leg-length equalization for patients undergoing primary total hip replacement. *The Journal of Arthroplasty*.

